# Surviving and Thriving With Cancer Using a Web-Based Health Behavior Change Intervention: Randomized Controlled Trial

**DOI:** 10.2196/jmir.3020

**Published:** 2014-02-24

**Authors:** Erin O'Carroll Bantum, Cheryl L Albright, Kami K White, Jeffrey L Berenberg, Gabriela Layi, Phillip L Ritter, Diana Laurent, Katy Plant, Kate Lorig

**Affiliations:** ^1^University of Hawaii Cancer CenterCancer Prevention & ControlHonolulu, HIUnited States; ^2^University of Hawaii at ManoaNursing and Dental HygieneHonolulu, HIUnited States; ^3^University of Hawaii Cancer CenterBiostatistics Shared ResourceHonolulu, HIUnited States; ^4^University of Hawaii Cancer CenterClinical and TranslationalHonolulu, HIUnited States; ^5^Tripler Army Medical CenterMedical OncologyHonolulu, HIUnited States; ^6^Stanford UniversitySchool of MedicineStanford Patient Education Research CenterPalo Alto, CAUnited States; ^7^National Council on AgingWashington, DCUnited States

**Keywords:** cancer survivors, online interventions, social support

## Abstract

**Background:**

Given the substantial improvements in cancer screening and cancer treatment in the United States, millions of adult cancer survivors live for years following their initial cancer diagnosis and treatment. However, latent side effects can occur and some symptoms can be alleviated or managed effectively via changes in lifestyle behaviors.

**Objective:**

The purpose of this study was to test the effectiveness of a six-week Web-based multiple health behavior change program for adult survivors.

**Methods:**

Participants (n=352) were recruited from oncology clinics, a tumor registry, as well as through online mechanisms, such as Facebook and the Association of Cancer Online Resources (ACOR). Cancer survivors were eligible if they had completed their primary cancer treatment from 4 weeks to 5 years before enrollment. Participants were randomly assigned to the Web-based program or a delayed-treatment control condition.

**Results:**

In total, 303 survivors completed the follow-up survey (six months after completion of the baseline survey) and participants in the Web-based intervention condition had significantly greater reductions in insomnia and greater increases in minutes per week of vigorous exercise and stretching compared to controls. There were no significant changes in fruit and vegetable consumption or other outcomes.

**Conclusions:**

The Web-based intervention impacted insomnia and exercise; however, a majority of the sample met or exceeded national recommendations for health behaviors and were not suffering from depression or fatigue at baseline. Thus, the survivors were very healthy and well-adjusted upon entry and their ability to make substantial health behavior changes may have been limited. Future work is discussed, with emphasis placed on ways in which Web-based interventions can be more specifically analyzed for benefit, such as in regard to social networking.

**Trial Registration:**

Clinicaltrials.gov NCT00962494; http://www.clinicaltrials.gov/ct2/show/NCT00962494 (Archived by WebCite at http://www.webcitation.org/6NIv8Dc6Q).

## Introduction

In the United States, there are currently more than 12 million people who have survived cancer [1]. The rise in this number over recent years is an accomplishment and can be attributed, in large part, to better screening and treatment. However, physical and psychological sequelae may persist after treatment leading to chronic latent side effects, with survivors reporting symptoms that occur 12 months or longer post treatment [2,3]. Cancer-related fatigue is the most persistent side effect regardless of tumor or treatment type [4]. Fatigue and other side effects (eg, edema, pain) can also lead to depression and anxiety [5]. After recovering from months of cancer therapy or surgeries, many cancer survivors want to not only return to their previous lifestyle but often have an interest in making positive changes in their health and quality of life. The point in time where this interest occurs has been coined a “teachable moment” [6,7] and can serve as an opportune time to introduce health behavior change strategies regardless of the type of cancer, stage at diagnosis, or the presence of late effects.

Having good social support has been linked to better health outcomes and quality of life in cancer survivors [8-11]. Despite these developments, health behavior change interventions for cancer survivors are seldom conducted in a group setting where social support from other survivors is encouraged. Interventions that include a social support component (eg, support groups) are much more prevalent, especially when the intervention is focused on psychosocial behavior change (eg, anxiety and depression) [12,13]. Such psychosocial interventions have shown that interacting with other cancer survivors is strongly related to better adjustment in terms of mood and quality of life [14]; thus, such interactions might also facilitate changes in health behaviors.

Since more than 83% of adults aged 50-64 and 56% of adults aged 65 and over have access to high-speed Internet connections via computers, laptops, and smartphones [15], and due to the somewhat anonymous nature of online cancer survivors’ support groups, online venues have become appealing to adult cancer survivors. However, the impact of Web-based multiple health behavior change interventions tailored to cancer survivors has been limited. Some of the first online research was conducted by Gustafson and colleagues [16] and suggested that computer-based programs focused on specific physical or psychological symptoms could lead to improvements in those symptoms. Some of the initial work consisted largely of transferring the content of workbooks to an online format with very little interactivity or interaction between survivors [17]. Some Web-based distress management interventions have been associated with benefit in terms of mood, perceived stress, and cancer-related trauma [13,18], while other interventions have similar results for both face-to-face and online interventions with the same content (eg, sexual counseling following prostate cancer treatment) [19], with one study demonstrating a negative impact [20] on distress and quality of life.

Recent Web-based interventions designed to change health behaviors vary substantially in terms of their design, online features, and length of follow-up. Such differences in online features can make comparisons between the results from these interventions difficult. Some Web-based interventions have a social networking component [18] while other interventions serve as more of an online repository for information [21]. The length of interventions also vary greatly, with some interventions being short and structured [21], while others are much longer [22]. In addition to differing lengths of intervention trials for cancer survivors, most trials include participants with one type of cancer [13,18,21,23], while few trials have brought together people with a range of cancer types [22]. There has been a limited amount of research focusing on people with a range of cancer types or multiple health behaviors.

The Chronic Disease Self-Management Program (CDSMP) was developed for people with chronic conditions and focuses on multiple health behaviors. Because the population is heterogeneous, there is no expectation that all participants will make similar behavior changes. This program has been shown to be effective across numerous health conditions (eg, diabetes, arthritis) and across multiple formats (face-to-face groups and online groups) [24,25]. More detailed description is provided in the Methods section, but key components include: Action Planning, Problem Solving, Decision Making, and Self-Tailoring. CDSMP is facilitated by two trained peer facilitators, one or both of whom have experienced a chronic disease. Facilitators read every post or “comment” made by a course participant, stimulate peer-to-peer interactions, and personally advise participants about how to set realistic, confidence-building health behavior goals. This format allows for peer interaction as well as structured facilitation. In order to examine whether the CDSMP intervention would also be effective for cancer survivors, CDSMP was adapted for cancer survivors to create the “Surviving and Thriving with Cancer” (STC) intervention.

The STC trial tested the effectiveness of a tailored Web-based intervention to encourage multiple health behavior changes in post-treatment adult cancer survivors. In order to maintain consistency with the CDSMP, in addition to being variables of importance for cancer survivors, diet, exercise, depression, and fatigue were chosen as our outcomes of interest. We hypothesized that participants in the STC treatment condition would show six-month improvements in psychosocial symptoms including fatigue, insomnia, and depression, and would also report eating significantly more servings per day of fruits and vegetables when compared with participants in the wait-list control condition. We also hypothesized that participants in STC would report significantly more minutes of physical activity per week compared to controls.

## Methods

### Participants

Eligibility requirements for the STC trial were intentionally broad and included age (18 years of age or older), completion of primary treatment at least four weeks prior, but not more than 5 years before joining the study, diagnosis with only one cancer and no recurrence, access to the Internet, and ability to read English.

### Recruitment

Potential participants were recruited via a number of different online and mailed strategies [26]. Recruitment at Stanford University was primarily conducted through online recruitment efforts, and in Hawaii, initial recruitment efforts focused on clinic-based recruitment in oncology offices on the island of Oahu, Tripler Army Medical Center (TAMC), and mailed recruitment letters to cancer survivors identified in the Tumor Registry at TAMC. However, in order to increase enrollment, recruitment methods were shifted to online nationwide recruitment via social networks used primarily by cancer survivors (Facebook, eg, “Throat Cancer Awareness”), Association of Cancer Online Resources (ACOR), and CURE print and digital magazine. Administrators of these various online sources were contacted and asked about willingness to post recruitment messages for their members. These messages invited interested people to learn more on the STC website. Approximately 60% (59.9%, 211/352) of the sample was recruited from these cancer-specific social media channels.

### Study Design

We used a randomized controlled delayed-treatment design (NCT00962494). Participants were directed to the STC website and screened for eligibility and then completed an online consent approved by the University of Hawaii and Stanford Institutional Review Boards (IRB). Participants from TAMC completed a mailed consent form that was approved by the military IRB. Once consented, participants completed an online baseline questionnaire and were then randomized to treatment or control status. Randomization was conducted on a group-by-group basis. Once 40 to 50 participants had completed their baseline questionnaire, they were numbered in the order of completion and then randomized, using a random number table, half to treatment and half to wait-list control. All participants received a US $10.00 Amazon voucher for completing each questionnaire.

### Intervention

The STC intervention was a six-week online workshop that was adapted from CDSMP [24], a patient education course adopting the underlying principle that people with similar health conditions can help each other improve their health behaviors. To create the STC program, a Web-based version of CDSMP was adapted to be more relevant for cancer survivors. The CDSMP’s modules on healthy eating were modified for cancer survivors living in Hawaii by adding foods that are commonly eaten in Hawaii, and modules on the changes in body, sleep, and other side effects associated with post-treatment recovery were added to the program.

**Figure 1:**
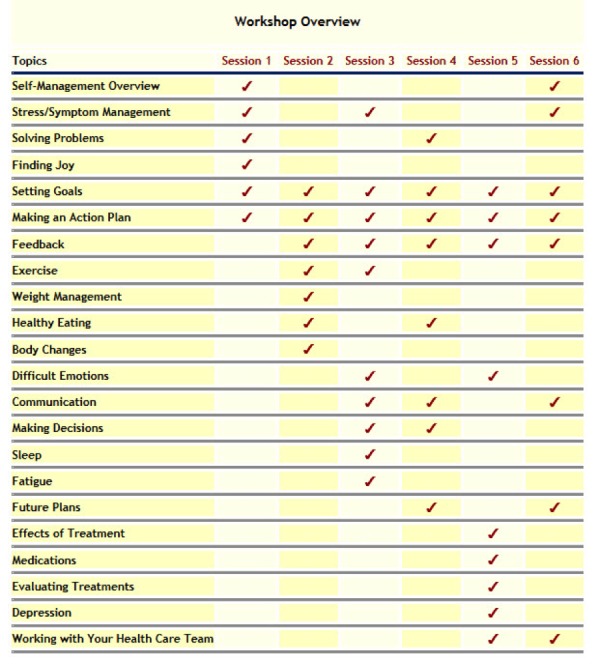
Topics included in the Surviving and Thriving with Cancer Intervention.

Each cohort (group) consisted of approximately 20-25 survivors, with a total of nine cohorts. Each session of the six-week course included approximately 30-35 webpages of didactic material (in the “Learning Center” of the STC*)* that is geared towards skills building, information about specific content, and the encouragement of weekly action plans to build self-efficacy. Examples of content include improving diet by making healthier food choices, increasing exercise, stress management via relaxation training, improving communication with health care providers, processing and communicating emotional experiences to people inside of one’s existing social network, as well as group members, and fatigue management. More details of weekly topics can be found in Figure 1. At the end of each weekly educational session, users were invited to identify a health behavior they would like to change and were guided, in both the didactic materials, as well as by facilitators on how to set realistic, achievable goals, which were called action plans. These weekly action plans were posted on the “Discussion Center” (see below) and facilitators provided feedback and help. Participants were prompted both in the middle and at the end of a given week, via an automated message, to update the group on their progress as well as provide feedback to other group members.

Each group had two facilitators who were cancer survivors. The facilitators went through intensive online training about both the content of the intervention materials as well as how to respond to users’ comments and goals. They were mentored by the principal investigators, who during the course of the intervention also read all posts and gave feedback and help to the facilitators as needed.

The STC intervention website contained numerous unique components. The most crucial components were the “Discussion Center”, “My Tools”, “Post Office”, and “Help”. The Discussion Center feature of the website is where social networking occurred and survivors were encouraged to provide feedback and encouragement to each other. This was accomplished in four threaded bulletin boards: action planning, problem solving, difficult emotions, and celebrations. As discussed above, these were seeded from the materials in the Learning Center. In addition, participants could post directly to any of the four bulletin boards at any time. The My Tools component of the program allowed participants to use tools (eg, exercise logs) to help continue to shape their behavior on an individual basis. They could also listen to relaxation exercises and find links to resources outside of this intervention. The Post Office component allowed participants to message each other individually, including emailing the facilitators. While facilitators, mentors, and principal investigators had access to all posted messages, they were not specifically monitored as a way to ensure some level of confidentiality. In the Help component, participants could contact one of the website or study administrators for assistance, look over a tutorial of the website, and read the informed consent.

### Data Collection

Survey data were collected at two time points: baseline and six months later. Although it is typical to survey participants immediately after completion of the intervention, the goal in waiting was to see if any changes following the intervention were maintained. The delayed treatment control condition received no information or materials over this period.

### Measures

Demographic and previous medical history items on the baseline questionnaire included: type and stage of cancer, date of diagnosis, course of treatment, co-morbidities, race/ethnicity, gender, marital status, and years of education. Measures were included to measure the following: fatigue, insomnia, exercise, fruit and vegetable intake, and depression. The Brief Fatigue Inventory (BFI) is a 15-item measure that was used to measure fatigue. It assesses both the severity of fatigue and the impact of fatigue on daily functioning during the last 24-hour period [27]. To measure insomnia, the 5-item validated Women’s Health Initiative Insomnia Rating Scale (WHIIRS) [28] was used. This measures how often, on a 5-point scale (from “no, not in the past 4 weeks” to “yes, 5 or more times a week), the participant experiences trouble falling or staying asleep. The Godin Exercise Questionnaire was used to assess minutes per week of exercise in the categories of mild, moderate, and vigorous [29]. The Block Food Frequency Questionnaire [30] was used to identify how many fruits and vegetables were eaten in the previous week and the number of servings were counted to represent the total fruit and vegetable consumption. The Patient Health Questionnaire (PHQ-8) was used to measure depression. This 8-item measure asks individuals to rate how much, on a 4-point scale (with options ranging from “not at all” to “nearly every day”), a given DSM diagnostic criteria for depression is perceived [31].

### Statistical Analysis

Baseline characteristics were reported as percentages for categorical variables and means and standard deviations for continuous variables. Differences between participants randomized to the control and intervention conditions were assessed using chi-square tests for categorical variables and *t* test for continuous variables. The primary analyses compared change from baseline to 6 months in the two conditions for the following outcome measures: fatigue, insomnia, minutes per week of physical activity (categorized as strenuous plus moderate aerobic, strenuous aerobic, moderate aerobic, mild aerobic, and stretching), servings of fruits and vegetables eaten per week, and depression. The physical activity outcome measures were transformed as (Y+1) to the 0.25 power, based on the Box-Cox method [31], to better meet model assumptions; all other outcomes were examined without transformation. Mixed linear models, including a random intercept term for each participant, were used to estimate and compare differences in outcomes over time between conditions. A second set of analyses was performed for the physical activity outcomes to address the many zero values reported by participants. A mixed-distribution model with random effects was used for these outcomes, simultaneously fitting a model for the probability of a value greater than zero and a model for the mean of values greater than zero [32]. The treatment effect was assessed by the *F* test of the fixed interaction parameter for time and intervention group. The effect size was computed by taking the differences between the means of the predicted values from the adjusted model at 6 months, divided by the standard deviation for the difference calculated from the within and between subject variance components. Models were adjusted for covariates selected a priori as likely to be related to the outcomes measures in this population. Adjustment variables included: age (continuous), race (white, nonwhite), gender, marital status (married, not married), smoking status (current, former, never), highest year of school completed (continuous), site of cancer diagnosis (breast, all others), cancer stage (in situ, stage 1, stage 2, stage 3, stage 4, unknown), and years since cancer was diagnosed. Subgroup analysis was performed by including a three-way interaction term between years since cancer diagnosis (≤2 or >2 years), condition group, and time, with all two-way interactions terms included. Model results are presented as means and 95% CIs of the predicted values obtained from the models.

Roughly 14% (13.9%, 49/352) of participants who were randomized did not provide any data at 6 months, which did not differ by condition (11.4%, 20/176 and 16.5%, 29/176) for control and intervention, respectively). To address attrition, correlates of attrition were identified using a logistic model regressing status (participants with data at 6 months vs participants with no 6-month data) onto baseline characteristics (same as adjustment variables listed above), condition group, and the presence of long term health conditions [including anxiety (yes, no), arthritis (yes, no), asthma (yes, no), back pain (yes, no), COPD (yes, no), depression (yes, no), diabetes (yes, no), high blood pressure (yes, no), heart disease (yes, no), sleep disorder (yes, no), and other (yes, no )], with a stepwise selection method.

Analyses were conducted using SAS, version 9.2. *P* values were two-sided and *P*<.05 was considered statistically significant.

## Results

### Participants

**Figure 2:**
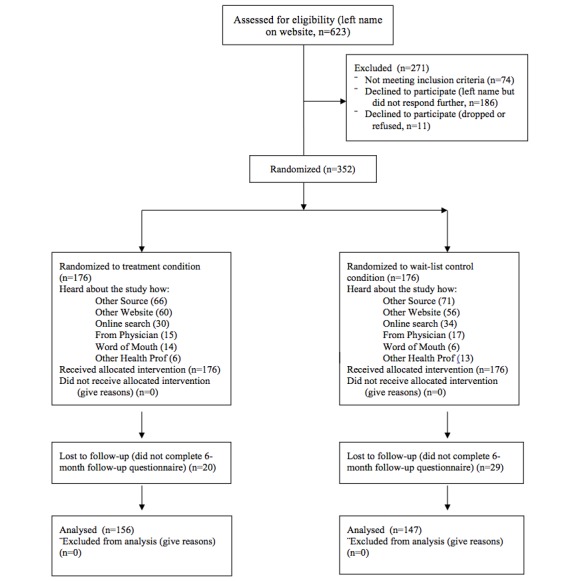
CONSORT recruitment diagram.

Recruitment strategies are discussed in detail elsewhere [26]. Briefly, 60% (59.9%, 211/352) of the interested participants were recruited from online social networking sites, the rest were recruited from physician offices, a tumor registry attached to Tripler Army Medical Center, and a survivorship clinic on Oahu. Figure 2 provides the CONSORT recruitment diagram for the study (also see Multimedia Appendix 1 for details). Overall, 623 people were screened for eligibility, 352 people completed baseline measures, and 303 completed follow-up measures (n*=*156 in treatment condition; n=147 in control condition; see Figure 2 for details). In testing for predictors of dropout, less education (OR 0.84, 95% CI 0.75-0.95, per one year increase) and having long-term back pain (OR 2.31, 95% CI 1.13-4.75) was associated with dropout between baseline and 6 month follow-up.

The majority of participants were Caucasian (87.2%, 307/352) and female (82.1%, 289/352), having a mean age of 51 years (SD 11.2) and mean education level of 16 years (SD 2.9); 47.4% (167/352) were diagnosed with breast cancer and another 12.8% (45/352) of participants were given either an ovarian or uterine cancer diagnosis. Baseline characteristics of participants in the control and intervention groups are shown in Table 1. With the exception of age, no significant differences were found among the two groups. Additionally, there were no significant differences between the control and treatment groups on all outcomes measures at baseline. Participants in both groups reported mild levels of fatigue (mean scores of about 40 on the BFI), insomnia (mean scores of 9.6 on the WHIIRS), and were engaging in moderate plus strenuous activity with median values of 1.5 to 2 hours per week. Participants also reported eating, on average, 23 servings of fruit and vegetables each week.

### Website Use

In regard to general use of the site, the mean number of sessions ever attended (logged on at least once) was 5.3 (SD 1.28) with the range being 0-6, and 67.0% (203/303) of participants attended all six sessions, with 86.8% (263/303) attending 4 or more sessions. There were 8016 total posts by treatment participants for an average of 46 posts per participant over the six-week intervention period.

### Health Behavior Impact

Results for changes in health behaviors/psychosocial outcomes are reported in Table 2. Significant interactions between condition group and time were found for insomnia, strenuous exercise, and stretching exercise. The intervention group experienced an improvement from baseline to 6 months compared to the control group: reduced insomnia (9.6 to 9.2 compared to 9.6 to 10.1, *P*=.03), increased strenuous exercise (32 to 51 min/wk compared to a steady 29 min/wk, *P*=.01), and increased stretching (31 to 46 min/wk compared to 26 to 25 min/wk, *P*=.01). In the subgroup analyses looking at differences between survivors with diagnoses ≤ 2 and >2 years prior to enrollment, there were no significant differences, although there were suggested trends seen for both insomnia (*P*=.07) and depression (*P*=.09), such that people who were greater than 2 years post treatment improved slightly more on those measures (data not shown).

**Table 1:** Baseline characteristics of study population.

Characteristic		Control group, (n=176), n (%)	Intervention group, (n=176), n (%)	*P*
Age, mean (SD)		49.3 (11.0)	52.4 (11.0)	.008
Female		148 (84.1)	141 (80.1)	.33
**Race**				.84
	Caucasian	150 (85.2)	157 (89.2)	
	Asian	9 (5.1)	8 (4.6)	
	African American	4 (2.3)	2 (1.1)	
	American Indian/Alaskan Native	3 (1.7)	2 (1.1)	
	Native Hawaiian/Pacific Islander	3 (1.7)	1 (0.6)	
	Other	7 (4.0)	6 (3.4)	
Married		122 (69.3)	109 (61.9)	.14
Highest year education attained, mean (SD)		16.5 (3.1)	16.3 (2.8)	.62
**Smoking status**				.43
	Current	4 (2.3)	7 (4.0)	
	Former	57 (32.4)	64 (36.4)	
	Never	115 (65.3)	105 (59.7)	
**Type of cancer diagnosed** ^a^				.70
	Breast	84 (47.7)	83 (47.2)	
	Endometrium/Uterine/Ovarian	23 (13.1)	22 (12.5)	
	Non-Hodgkins Lymphoma	13 (7.4)	7 (4.0)	
	Colorectal	11 (6.5)	11 (6.5)	
	Lung	7 (4.0)	8 (4.6)	
	Thyroid	6 (3.4)	8 (4.6)	
	Oral	6 (3.4)	5 (2.8)	
**Stage of cancer**				.96
	In situ	9 (5.1)	7 (4.0)	
	Stage 1	45 (25.6)	45 (25.6)	
	Stage 2	52 (29.6)	55 (31.3)	
	Stage 3	37 (21.0)	41 (23.3)	
	Stage 4	16 (9.1)	13 (7.4)	
	Unknown	17 (9.7)	15 (8.5)	
Number of years since cancer diagnosed, mean (SD)		2.5 (1.3)	2.4 (1.4)	.41
Number of years since treatment completed, mean (SD)		1.9 (1.2)	1.7 (1.2)	.09
**Prevalence of long-term health conditions**				
	High blood pressure	31 (17.6)	34 (19.3)	.68
	Depression	32 (18.2)	29 (16.5)	.67
	Back pain	25 (14.2)	29 (16.5)	.55
	Anxiety	29 (16.5)	23 (13.1)	.37
	Arthritis	18 (10.2)	27 (15.3)	.15
	Sleep disorder	18 (10.2)	12 (6.8)	.25
	Asthma	13 (7.4)	13 (7.4)	1.0
	Diabetes	9 (5.1)	11 (6.3)	.65
	Heart disease	3 (1.7)	8 (4.6)	.13
	Emphysema, COPD, chronic bronchitis	3 (1.7)	5 (2.8)	.72
	Other	39 (22.2)	39 (22.2)	1.0

^a^Sites also reported were oral cavity (n=11), soft tissue (n=11), testicular (n=10), kidney and renal (n=10), and other [n=26, including brain (n=5), prostate (n=4), eye (n=3)].

**Table 2:** Mean (95% CI)^a^ of outcome measures from baseline to 6 months by condition group.

Outcome measures	Control group, mean (95% CI)		Intervention group, mean (95% CI)		*P* ^b^	Effect size Month 6^c^
	Baseline (n=176)	Month 6 (n=156)	Baseline (n=176)	Month 6 (n=147)		
Fatigue (BFI^d^)	40.8 (38.9-42.8)	40.7 (38.7-42.8)	39.0 (37.0-40.9)	36.4 (34.2-38.5)	.56	.17
Insomnia (WHIIRS^e^)	9.6 (9.1-10.1)	10.1 (9.6-10.7)	9.6 (9.1-10.1)	9.2 (8.7-9.8)	.03	.20
Depression (PHQ^f^)	7.7 (7.0-8.3)	7.1 (6.4-7.7)	6.5 (5.9-7.1)	6.1 (5.4-6.7)	.69	.19
Fruit/vegetable intake, times/week	22.7 (21.4-24.1)	23.2 (21.7-24.7)	24.3 (23.1-25.6)	25.9 (24.6-27.3)	.24	.21
Strenuous or moderate aerobic exercise, min/week	86.0 (72.3-99.7)	96.2 (79.9-112)	106 (91.1-120)	137 (119-155)	.45	.29
Strenuous aerobic exercise, min/week	29.0 (22.5-35.5)	28.9 (21.8-36.0)	32.0 (25.5-38.5)	50.8 (40.7- 60.9)	.01	.36
Moderate aerobic exercise, min/week	37.0 (30.9-43.2)	45.3 (37.5-53.0)	49.0 (42.2-55.7)	54.1 (46.5- 61.7)	.49	.10
Mild aerobic exercise, min/week	58.9 (51.5-66.2)	65.0 (56.5-73.6)	56.1 (48.9-63.3)	74.1 (64.2-84.1)	.28	.10
Stretching min/week	25.9 (21.3-30.4)	24.7 (20.0-29.5)	30.5 (25.1-35.8)	45.7 (38.1-53.4)	.01	.12

^a^Adjusted for age, race, sex, marital status, smoking status, education, years since diagnosis, site of cancer diagnosis, cancer stage. For outcomes of fatigue, insomnia, depression, and fruit/vegetable intake, means and 95% CIs were computed on the predicted values from the model. For outcomes of physical activity, means and 95% CIs were computed on the back-transformed predicted values (Y^4^-1), where Y represented the predicted values from the model.

^b^Treatment effect was assessed by the *F* test of the fixed 2-way interaction parameter for time and condition group.

^c^Calculated by taking the differences of the means at 6 months predicted from the model, including adjustment factors, divided by the standard deviation for the difference computed from the within and between subject variance components.

^d^BFI: Brief Fatigue Inventory

^e^WHIIRS: Women’s Health Initiative Insomnia Rating Scale

^f^PHQ: Patient Health Questionnaire

## Discussion

### Principal Findings

Participants in the treatment condition had significant reductions in insomnia and engaged in more strenuous and stretching exercises than those in the control condition. There is an established link between sleep disturbance and inflammation, which can be related to both cancer and depression [33], so impacting insomnia is a relevant finding. There is only one other known Web-based exercise and diet intervention for adult cancer survivors [34]. Although outcomes of that study are not yet available, we have found the current system usable and the intervention feasible. In regard to face-to-face interventions to impact exercise for cancer survivors, these have been demonstrated to be effective [35-37], often times with larger effect sizes than were demonstrated in this trial. This is crucial because Web-based interventions have relevance for people who have physical limitations or are not near facilities that could offer face-to-face interventions. Health behavior change interventions are relevant for cancer survivors, so continuing to test and refine interventions is imperative in the area of cancer survivorship.

### Limitations

There are some limitations of the current study that should be noted. We measured health behaviors via self-report and there may have been over/underestimations of the dietary intake of fruits and vegetables, as well as physical activity, due to social desirability or recall bias. Due to significant economic, logistical, and noncompliance issues that can occur when nationwide online studies use objective measures for physical activity (eg, accelerometer) or telephone interviews for dietary intake (eg, 24-hour recall), this study was not able to use these types of assessments. That being said, self-reported health behaviors are commonly used for both Web-based and face-to-face trials and for several national health risk behavior surveys conducted by the NIH and CDC. Although the study focused on multiple outcomes, we did not adjust the significance level for multiple comparisons due to the exploratory nature of the analyses.

Our sample was well-educated and because more than half were recruited from various Internet sites, they had high levels of computer literacy and, thus, might be more familiar with posting their personal experiences on bulletin boards so others could comment on their success or lack thereof. Participants were not recruited or screened for entry based on specific inclusion or exclusion criteria for any specific health behavior (eg, low levels of physical activity or high levels of fatigue as criteria for eligibility) or for their inherent motivation/need to change all of the health behaviors addressed in the intervention. While this could have resulted in recruiting persons who were the most interested or more in need of changing a specific health behavior, in our study, it resulted in participants who were healthy, very well-adjusted, with little to no need (according to current recommendations) for significant changes in their health behaviors. At enrollment, their exercise and eating behaviors (in regard to intake of fruits and vegetables) were better than seen in national surveys, given that only 59% of average Americans eat the recommended 2.5 servings of vegetables per day and 42% eat the recommended 2.5 servings of fruits per day [38]. Scores on our depression measure indicate that participants, as a whole, had no concerns with depression. This leads to the question of whether we recruited survivors who were in need of support to improve multiple unhealthy habits, as well as whether this is one of the primary reasons that significant changes were not seen on many of the outcomes of interest (fatigue, depression, and increases in fruit and vegetable intake). The participants could also choose the behavior they wanted to change, regardless of their baseline level of that behavior or “need” to improve it. These factors could have contributed to the lack of significant change over the six-month period on some of the other outcome measures. In addition, when doing a population study where people enter with different concerns and a large range of scores on baseline measures, effect sizes can be muted. With a larger sample size, sensitivity analyses including only people who were not engaging in the health behaviors of interest at baseline could be explored. Future research could take into consideration these issues.

Another potential limitation is in regard to the lack of participants with a range of cancer types. As has been the case in the past and was the case with our study, the sample included a large percentage of female breast cancer survivors (47% of the sample), suggesting that the sample was more homogenous and perhaps the findings are less generalizable to people with other types of cancer. Future efforts for this to be more balanced are important and will be made in upcoming work. Although efforts were made to recruit people who would be more representative of cancer survivors as a whole in regards to gender, ethnicity, and cancer type, those efforts fell short in this study and continued efforts will be made.

### Conclusions

Web-based interventions provide the ability to more fully understand the intervention aspects that are of most interest to cancer survivors, and with many of these interventions including social networking features, to understand the ways in which people interact and how that might be related to outcomes. People who have survived cancer clearly valued the social networking aspects of the STC site. There were multiple social networking components, such as webmail and numerous different discussion boards, so additional analyses could be conducted to understand what might be most important to the participants in terms of social networking. Understanding more about who people interacted with, as well as the content of those interactions, provides a foundation to more fully understand the ways in which people connect and how those connections matter in these sorts of interventions. Continued inclusion of social networking/online support in these types of interventions, as well as data collection on usage, is encouraged. Better understanding how the components included are used could also be a way to identify potent features of the intervention. It is important to note, though, that there could be synergistic effects that are difficult to capture technically when isolating components of interest. In conclusion, the Thriving and Surviving with Cancer intervention has been proven a relative success and additional efforts to understand what components are related to the most success could help further develop this, or any, Web-based intervention program.

## Multimedia Appendix 1

CONSORT-EHEALTH checklist V1.6.2 [39].

## Abbreviations

ACOR: Association of Cancer Online Resources
BFI: Brief Fatigue Inventory
CDSMP: Chronic Disease Self-Management Program
PHQ: Patient Health Questionnaire
STC: Surviving and Thriving with Cancer
TAMC: Tripler Army Medical Center
WHIIRS: Women’s Health Initiative Insomnia Rating Scale
