# Supplementary material for: Surviving and Thriving With Cancer Using a Web-Based Health Behavior Change Intervention: Randomized Controlled Trial
Source: J Med Internet Res. 2014 Feb 24;16(2):e54. doi: 10.2196/jmir.3020 (PMC3961702; doi:10.2196/jmir.3020)
Supplement: Supplementary file 1 [file jmir_v16i2e54_app1.pdf]

CONSORT-EHEALTH Checklist V1.6.2 Report

(based on CONSORT-EHEALTH V1.6), available at [http://tinyurl.com/consort-ehealth-v1-6].

3020

Date completed

2/19/2014 17:33:41

by

Bantum

Surviving and Thriving With Cancer: A Randomized-Controlled Trial of Online Health Behavior Change

TITLE

1a-i) Identify the mode of delivery in the title

The title contains the word "online" but also contains "randomized-controlled trial"

1a-ii) Non-web-based components or important co-interventions in title

Yes- the title says the word "cancer" and "health behavior change"

1a-iii) Primary condition or target group in the title

"cancer"

ABSTRACT

1b-i) Key features/functionalities/components of the intervention and comparator in the METHODS section of the ABSTRACT

"test the effectiveness of a six-week online multiple health behavior program for adult survivors"

1b-ii) Level of human involvement in the METHODS section of the ABSTRACT

"Participants (n = 352) were recruited from oncology clinics, a tumor registry, as well as through online mechanisms, such as Facebook and Association of Cancer Online Resources (ACOR). "

1b-iii) Open vs. closed, web-based (self-assessment) vs. face-to-face assessments in the METHODS section of the ABSTRACT

The abstract doesn't speak to whether data was collected online.

1b-iv) RESULTS section in abstract must contain use data

"303 survivors completed the follow-up survey (6 months after completion of the baseline survey) and participants in the online intervention condition had significantly greater reductions in insomnia, and greater increases in minutes per week of vigorous exercise, and stretching compared to controls"

1b-v) CONCLUSIONS/DISCUSSION in abstract for negative trials

"The online intervention impacted insomnia and exercise; however, a majority of the sample met or exceeded national recommendations for health behaviors and were not suffering from depression or fatigue at baseline"

INTRODUCTION

2a-i) Problem and the type of system/solution

"However, physical and psychological sequelae may persist after treatment leading to chronic, latent side effects, with survivors reporting symptoms that occur 12-months or longer post treatment [2,3]. Cancer-related fatigue is the most persistent side effect regardless of tumor or treatment type [4]. Fatigue and other side-effects (e.g., edema, pain) can also lead to depression and anxiety [5]. After recovering from months of cancer therapy or surgeries, many cancer survivors want to not only return to their previous lifestyle but often have an interest in making positive changes in their health and quality of life. The point in time where this interest occurs has been coined a “teachable moment” [6,7] and can serve as an opportune time to introduce health behavior change strategies regardless of the type of cancer, stage at diagnosis, or the presence of late effects."

"Despite these developments, health-behavior change interventions for healthy cancer survivors are seldom conducted in a group setting where social support from other survivors is encouraged. Interventions that include a social support component (e.g., support groups) are much more prevalent, especially when the intervention is focused on psychosocial behavior change (i.e., anxiety and depression) [12,13]."

2a-ii) Scientific background, rationale: What is known about the (type of) system

"Since over 83% of adults aged 50-64 and 56% of adults aged 65 and over have access to high-speed Internet connections via computers/laptops/Smartphones [15] and due to the somewhat anonymous nature of online cancer survivors’ support groups, online venues have become appealing to adult cancer survivors. However, the impact of online multiple health behavior change interventions tailored to cancer survivors has been limited. Some of the first online research was conducted by Gustafson and colleagues [16] and suggested that computer-based programs focused on specific physical or psychological symptoms could lead to improvements in those symptoms. Some of the initial work consisted largely of transferring the content of workbooks to an online format with very little interactivity or interaction between survivors [17]. Some online distress management interventions have been associated with benefit in terms of mood, perceived stress, and cancer-related trauma [13, 18], while other interventions have similar results for both face-to-face and online interventions with the same content (e.g., sexual counseling following prostate cancer treatment) [19], with one study demonstrating a negative impact [20] on distress and quality of life.
Recent Internet interventions designed to change health behaviors vary substantially in terms of their design, online features and length of follow-up. Such differences in online features can make comparisons between the results from these interventions difficult. Some online interventions have a social networking component [18] while other interventions serve as more of an online repository for information [21]. The length of interventions also vary greatly, with some interventions being short and structured [21], while others are much longer [23]. In addition to differing lengths of intervention trials for cancer survivors, most trials include participants with one type of cancer [13,18,21] while few trials have brought together people with a range of cancer types [23]. There has been a limited amount of research focusing on people with a range of cancer types or multiple health behaviors.
The Chronic Disease Self-Management Program (CDSMP) was developed for people with chronic conditions and focuses on multiple health behaviors. Because the population is heterogeneous there is no expectation that all participants will make similar behavior changes. This program has been shown to be effective across numerous health conditions (e.g., diabetes, arthritis) and across multiple formats (face-to-face groups and online groups) [24,25]. More detailed description is provided in the methods section, but key components include: Action Planning, Problem Solving, Decision Making and Self-Tailoring. CDSMP is facilitated by two trained peer facilitators, one or both of whom have experienced a chronic disease. Facilitators read every post or “comment” made by a course participant, stimulate peer-to-peer interactions, and personally advise participants about how to set realistic, confidence building health behavior goals. This format allows for peer interaction as well as structured facilitation."

METHODS

3a) CONSORT: Description of trial design (such as parallel, factorial) including allocation ratio

". In order to examine whether the CDSMP intervention would also be effective for cancer survivors, CDSMP was adapted for cancer survivors, creating Surviving and Thriving with Cancer (STC).
The STC trial tested the efficacy of a tailored online intervention to encourage multiple health behavior changes in post treatment adult cancer survivors. In order to maintain consistency with the CDSMP, in addition to being variables of importance for cancer survivors, diet, exercise, depression, and fatigue were chosen as our outcomes of interest. We hypothesized that participants in the STC treatment condition would show six-month improvements in psychosocial symptoms including fatigue, insomnia, and depression, and would also report eating significantly more servings per day of fruits and vegetables when compared with participants in the wait-list control condition. We also hypothesized that participants in STC would report significantly more minutes of physical activity per week compared to controls."

3b) CONSORT: Important changes to methods after trial commencement (such as eligibility criteria), with reasons

"Recruitment at Stanford was primarily conducted through online recruitment efforts, and in Hawai'i, initial recruitment efforts focused on clinic-based recruitment in oncology offices on the island of Oahu, HI, Tripler Army Medical Center (TAMC), and mailed recruitment letters to cancer survivors identified in the Tumor Registry at TAMC. However, in order to increase enrollment, recruitment methods were shifted to online nationwide recruitment via social networks used primarily by cancer survivors (Facebook, e.g., “Throat Cancer Awareness”), Association of Cancer Online Resources (ACOR), and CURE print and digital magazine)."

3b-i) Bug fixes, Downtimes, Content Changes

"The STC intervention was a 6-week online workshop that was adapted from the Chronic Disease Self-Management Program (CDSMP) [24]. CDSMP is a patient education course adopting the underlying principle that people with similar health conditions can help each other improve their health behaviors. To create the STC program, an online version of CDSMP was adapted to be more relevant for cancer survivors. The CDSMP’s modules on healthy eating were modified for cancer survivors living in Hawaii by adding foods that are commonly eaten in Hawaii, and modules on the changes in body, sleep, and other side effects associated with post- treatment recovery were added to the program."

4a) CONSORT: Eligibility criteria for participants

"Eligibility requirements for the STC trial were intentionally broad and included age (18 years of age or older), completion of primary treatment at least four weeks prior to joining the study but not more than 5 years post treatment, diagnosis with only one cancer and no recurrence, access to the Internet, and ability to read English."

4a-i) Computer / Internet literacy

"access to the Internet, and ability to read English."

4a-ii) Open vs. closed, web-based vs. face-to-face assessments:

". Participants were directed to the website and screened for eligibility and then completed an on-line consent approved by the University of Hawaii and Stanford IRBs. Participants from TAMC completed a mailed consent form that was approved by the military IRB. Once consented, participants completed an on-line baseline questionnaire and were then randomized to treatment or control status. Randomization was conducted on a group-by-group basis. Once 40 to 50 participants had completed their baseline questionnaire they were numbered in the order of completion and then randomized, using a random number table, half to treatment and half to wait-list control. All participants received a $10.00 Amazon voucher for completing each questionnaire."

4a-iii) Information giving during recruitment

"Participants were directed to the website and screened for eligibility and then completed an on-line consent approved by the University of Hawaii and Stanford IRBs."

4b) CONSORT: Settings and locations where the data were collected

The paper does not talk about where data was collected, aside from stating this was done "online."

4b-i) Report if outcomes were (self-)assessed through online questionnaires

"Demographic and previous medical history items on the baseline questionnaire included: type and stage of cancer, date of diagnosis, course of treatment, co-morbidities, race/ethnicity, gender, marital status, and years of education. The following measures were included: Fatigue. The Brief Fatigue Inventory (BFI) is a 15-item measure that was used to measure fatigue. It assesses both the severity of fatigue and the impact of fatigue on daily functioning during the last 24-hour period [27]. Insomnia. To measure insomnia the 5-item, validated Women’s Health Initiative Rating Scale (WHIRS) [28] was used. This measures how often on a 5-point scale (from “no, not in the past 4 weeks” to “yes, 5 or more times a week) participant experiences trouble falling or staying asleep. Exercise. The Godin Exercise Questionnaire was used to assess minutes per week of exercise in the categories of mild, moderate, and vigorous [29]. Fruit and Vegetable Intake. The Block Food Frequency Questionnaire [30] was used to identify how many fruits and vegetables were eaten in the previous week and the number of servings were counted to represent the total fruit and vegetable consumption. Depression. The Patient Health Questionnaire (PHQ-8) was used to measure depression. This 8-item measure asks individuals to rate how much, on a 4-point scale (with options ranging from “not at all” to “nearly every day”), a given DSM diagnostic criteria for depression is perceived [31]."

4b-ii) Report how institutional affiliations are displayed

5) CONSORT: Describe the interventions for each group with sufficient details to allow replication, including how and when they were actually administered

5-i) Mention names, credential, affiliations of the developers, sponsors, and owners

All of the author and funding information is included in the paper. There were no outside developers used.

5-ii) Describe the history/development process

This was an existing intervention, although as previously described we did detail the aspects of the intervention that changed for relevance to this population.

5-iii) Revisions and updating

This intervention did not have changing content, aside from the details posted by the participants: "CDSMP is facilitated by two trained peer facilitators, one or both of whom have experienced a chronic disease. Facilitators read every post or “comment” made by a course participant, stimulate peer-to-peer interactions, and personally advise participants about how to set realistic, confidence building health behavior goals."

"This feature of the website is where social networking occurred and survivors were encouraged to provide feedback and encouragement to each other. This was accomplished in four threaded bulletin boards, action planning, problem solving, difficult emotions, and celebrations. As discussed above these were seeded from the Learning Center. In addition, participants could post directly to any of the four bulletin boards at any time."

"The Post Office component allowed participants to message each other individually, including emailing the facilitators. While facilitators, mentors, and principal investigators had access to all posted messages, they were not specifically monitored as a way to ensure some level of confidentiality."

5-iv) Quality assurance methods

This was not specifically detailed in the manuscript, although this was the role of the facilitators, and this could perhaps, be inferred from previously described information.

5-v) Ensure replicability by publishing the source code, and/or providing screenshots/screen-capture video, and/or providing flowcharts of the algorithms used

These details were not included.

5-vi) Digital preservation
This site is no longer active.
5-vii) Access
"Participants were prompted both in the middle and at the end of a given week, via an automated message, to update the group on their progress as well as provide feedback to other group members."

"All participants received a $10.00 Amazon voucher for completing each questionnaire."
5-viii) Mode of delivery, features/functionalities/components of the intervention and comparator, and the theoretical framework
"The intervention contained numerous unique components. The most crucial components were as follows:
Discussion Center. This feature of the website is where social networking occurred and survivors were encouraged to provide feedback and encouragement to each other. This was accomplished in four threaded bulletin boards, action planning, problem solving, difficult emotions, and celebrations. As discussed above these were seeded from the Learning Center. In addition, participants could post directly to any of the four bulletin boards at any time.
My Tools. This component of the program allowed participants to use tools (i.e., exercise logs) to help continue to shape their behavior on an individual basis. They could also listen to relaxation exercises and find links to resources outside of this intervention.
Post Office. The Post Office component allowed participants to message each other individually, including emailing the facilitators. While facilitators, mentors, and principal investigators had access to all posted messages, they were not specifically monitored as a way to ensure some level of confidentiality.
Help. In the Help component, participants could contact one of the website or study administrators for assistance, look over a tutorial of the website, and read the informed consent.
Data Collection
Survey data were collected at two time points, baseline and six months later. Although it is typical to survey participants immediately after completion of the intervention, the goal in waiting was to see if any changes following the intervention were maintained. The delayed treatment control condition received no information or materials over this period."
5-ix) Describe use parameters
"Each session of the 6-week course included approximately 30-35 web pages of didactic material (in the Learning Center of the STC) that is geared towards skills building, information about specific content, and the encouragement of weekly action plans to build self-efficacy. Examples of content include improving diet by making healthier food choices, increasing exercise, stress management via relaxation training, improving communication with health care providers, processing and communicating emotional experiences to people inside of one's existing social network, as well as group members, and fatigue management. More details of weekly topics can be found in Figure 1. At the end of each weekly educational session, users were invited to identify a health behavior they would like to change and were guided, in both the didactic materials, as well as by facilitators on how to set realistic, achievable goals, which were called action plans. These weekly action plans were posted on the Discussion Center (see below) and facilitators provided feedback and help. Participants were prompted both in the middle and at the end of a given week, via an automated message, to update the group on their progress as well as provide feedback to other group members."
5-x) Clarify the level of human involvement
This was discussed in a number of places, but most directly in "Each group had two facilitators who were cancer survivors. The facilitators went through intensive on-line training about both the content of the intervention materials as well as how to respond to users' comments/goals. They were mentored by the principal investigators, who during the course also read all posts and gave feedback and help to the facilitators as needed."
5-xi) Report any prompts/reminders used
"At the end of each weekly educational session, users were invited to identify a health behavior they would like to change and were guided, in both the didactic materials, as well as by facilitators on how to set realistic, achievable goals, which were called action plans. These weekly action plans were posted on the Discussion Center (see below) and facilitators provided feedback and help. Participants were prompted both in the middle and at the end of a given week, via an automated message, to update the group on their progress as well as provide feedback to other group members."
5-xii) Describe any co-interventions (incl. training/support)
"This component of the program allowed participants to use tools (i.e., exercise logs) to help continue to shape their behavior on an individual basis. They could also listen to relaxation exercises and find links to resources outside of this intervention."
6a) CONSORT: Completely defined pre-specified primary and secondary outcome measures, including how and when they were assessed
"We hypothesized that participants in the STC treatment condition would show six-month improvements in psychosocial symptoms including fatigue, insomnia, and depression, and would also report eating significantly more servings per day of fruits and vegetables when compared with participants in the wait-list control condition. We also hypothesized that participants in STC would report significantly more minutes of physical activity per week compared to controls."
6a-i) Online questionnaires: describe if they were validated for online use and apply CHERRIES items to describe how the questionnaires were designed/deployed
We did not describe whether these were validated for online use.
6a-ii) Describe whether and how "use" (including intensity of use/dosage) was defined/measured/monitored
This was not discussed in detail, although the following was reported: "In regards to general use of the site, the mean number of sessions ever attended (logged on at least once) was 5.3, and 67% of participants attended all six sessions, with 87% attending 4 or more sessions. There were 8,016 total posts by treatment participants for an average of 46 posts per participant over the six-week intervention period."
6a-iii) Describe whether, how, and when qualitative feedback from participants was obtained
If qualitative feedback was part of this intervention it would be important to include, although it wasn't included in this intervention.
6b) CONSORT: Any changes to trial outcomes after the trial commenced, with reasons
This did not occur.
7a) CONSORT: How sample size was determined
7a-i) Describe whether and how expected attrition was taken into account when calculating the sample size
The original power analysis was not discussed.
7b) CONSORT: When applicable, explanation of any interim analyses and stopping guidelines
It is unclear what "interim analyses" refers to, although guidelines were not stopped at any point.
8a) CONSORT: Method used to generate the random allocation sequence
N/A
8b) CONSORT: Type of randomisation; details of any restriction (such as blocking and block size)
"Once consented, participants completed an on-line baseline questionnaire and were then randomized to treatment or control status. Randomization was conducted on a group-by-group basis. Once 40 to 50 participants had completed their baseline questionnaire they were numbered in the order of completion and then randomized, using a random number table, half to treatment and half to wait-list control. All participants received a $10.00 Amazon voucher for completing each questionnaire."

9) CONSORT: Mechanism used to implement the random allocation sequence (such as sequentially numbered containers), describing any steps taken to conceal the sequence until interventions were assigned
N/A
10) CONSORT: Who generated the random allocation sequence, who enrolled participants, and who assigned participants to interventions
N/A
11a) CONSORT: Blinding - If done, who was blinded after assignment to interventions (for example, participants, care providers, those assessing outcomes) and how
11a-i) Specify who was blinded, and who wasn't
People were not blinded once randomized.
11a-ii) Discuss e.g., whether participants knew which intervention was the "intervention of interest" and which one was the "comparator"
This was a wait-list control study, so participants were immediately aware if they would receive the intervention immediately or need to wait.
11b) CONSORT: If relevant, description of the similarity of interventions
N/A
12a) CONSORT: Statistical methods used to compare groups for primary and secondary outcomes
"Baseline characteristics were reported as percentages for categorical variables and means and standard deviations for continuous variables. Differences between participants randomized to the control and intervention conditions were assessed using $\chi 2$ tests for categorical variables and t-test for continuous variables. The primary analyses compared change from baseline to 6 months in the two conditions for the following outcome measures: fatigue, insomnia, minutes per week of physical activity (categorized as strenuous plus moderate aerobic, strenuous aerobic, moderate aerobic, mild aerobic, and stretching), servings of fruits and vegetables eaten per week, and depression. The physical activity outcome measures were transformed as (Y+1) to the 0.25 power, based on the Box-Cox method [31], to better meet model assumptions; all other outcomes were examined without transformation. Mixed linear models, including a random intercept term for each participant, were used to estimate and compare differences in outcomes over time between conditions. A second set of analyses was performed for the physical activity outcomes to address the many zero values reported by participants. A mixed-distribution model with random effects was used for these outcomes, simultaneously fitting a model for the probability of a value greater than zero and a model for the mean of values greater than zero [32]. The treatment effect was assessed by the F test of the fixed interaction parameter for time and intervention group. The effect size was computed by taking the differences between the means of the predicted values from the adjusted model at 6 months, divided by the standard deviation for the difference calculated from the within and between subject variance components. Models were adjusted for covariates selected a priori as likely to be related to the outcomes measures in this population. Adjustment variables included: age (continuous), race (White, non White), gender, marital status (married, not married), smoking status (current, former, never), highest year of school completed (continuous), site of cancer diagnosis (breast, all others), cancer stage (in situ, stage 1, stage 2, stage 3, stage 4, unknown), and years since cancer was diagnosed. Subgroup analysis was performed by including a three-way interaction term between years since cancer diagnosis (≤2 or >2 years), condition group, and time, with all two-way interactions terms included. Model results are presented as means and 95% CIs of the predicted values obtained from the models."
12a-i) Imputation techniques to deal with attrition / missing values
"Roughly 14% of participants who were randomized did not provide any data at 6 months, which did not differ by condition (11% and 16% for control and intervention, respectively). To address attrition, correlates of attrition were identified using a logistic model regressing status (participants with data at 6 months vs. participants with no 6 month data) onto baseline characteristics (same as adjustment variables listed above), condition group, and the presence of long term health conditions (including anxiety (yes, no), arthritis (yes, no), asthma (yes, no), back pain (yes, no), COPD (yes, no), depression (yes, no), diabetes (yes, no), high blood pressure (yes, no), heart disease (yes, no), sleep disorder (yes, no), and other (yes, no)), with a stepwise selection method."
12b) CONSORT: Methods for additional analyses, such as subgroup analyses and adjusted analyses
"Subgroup analysis was performed by including a three-way interaction term between years since cancer diagnosis (≤2 or >2 years), condition group, and time, with all two-way interactions terms included. Model results are presented as means and 95% CIs of the predicted values obtained from the models."

RESULTS

13a) CONSORT: For each group, the numbers of participants who were randomly assigned, received intended treatment, and were analysed for the primary outcome
This is detailed in the consort figure, which is Figure 2 of the manuscript.

13b) CONSORT: For each group, losses and exclusions after randomisation, together with reasons
This is shown in the consort figure.
13b-i) Attrition diagram
This is demonstrated in the consort figure.
14a) CONSORT: Dates defining the periods of recruitment and follow-up
The recruitment dates are not specified.
14a-i) Indicate if critical "secular events" fell into the study period

N//A, as this did not occur.

14b) CONSORT: Why the trial ended or was stopped (early)

N/A. The trial was not stopped early

15) CONSORT: A table showing baseline demographic and clinical characteristics for each group

This is table 1 in our manuscript.

15-i) Report demographics associated with digital divide issues

This is not reported or discussed.

16a) CONSORT: For each group, number of participants (denominator) included in each analysis and whether the analysis was by original assigned groups

16-i) Report multiple "denominators" and provide definitions

This is included in each table detailing results.

16-ii) Primary analysis should be intent-to-treat

All participants stayed in their originally assigned condition.

17a) CONSORT: For each primary and secondary outcome, results for each group, and the estimated effect size and its precision (such as 95% confidence interval)

Effect size and precision is included in each analysis.

17a-i) Presentation of process outcomes such as metrics of use and intensity of use

This was not included in the manuscript.

17b) CONSORT: For binary outcomes, presentation of both absolute and relative effect sizes is recommended

N/A

18) CONSORT: Results of any other analyses performed, including subgroup analyses and adjusted analyses, distinguishing pre-specified from exploratory

"In the subgroup analyses looking at differences between survivors with diagnoses ≤ and > 2 years prior to enrollment, there were no significant differences, although there were suggested trends seen for both insomnia ($P = .07$) and depression ($P = .09$), such that people who were greater than 2 years post treatment improved slightly more on those measures (data not shown)."

18-i) Subgroup analysis of comparing only users

N/A

19) CONSORT: All important harms or unintended effects in each group

No unintended effects were found or documented.

19-i) Include privacy breaches, technical problems

This did not occur.

19-ii) Include qualitative feedback from participants or observations from staff/researchers

This was not included.

DISCUSSION

20) CONSORT: Trial limitations, addressing sources of potential bias, imprecision, multiplicity of analyses

20-i) Typical limitations in ehealth trials

There are some limitations of the current study that should be noted. We measured health behaviors via self-report and there may have been over/under estimations of the dietary intake of fruits and vegetables, as well as physical activity, due to social desirability or recall bias. Due to significant economic, logistical, and noncompliance issues that can occur when nationwide online studies use objective measures for physical activity (i. e., accelerometer) or telephone interviews for dietary intake (i.e., 24 hour recall), this study was not able to use these types of assessments. That being said, self-reported health behaviors are commonly used for both online and face-to-face trials and for several national health risk behavior surveys conducted by the NIH and CDC. Although the study focused on multiple outcomes, we did not adjust the significance level for multiple comparisons due to the exploratory nature of the analyses."

21) CONSORT: Generalisability (external validity, applicability) of the trial findings

21-i) Generalizability to other populations

"Although outcomes of that study are not yet available, we have found the current system usable and the intervention feasible. In regards to face-to-face interventions to impact exercise for cancer survivors, these have been demonstrated to be effective [36-38], often times with larger effect sizes than were demonstrated in this trial. This is crucial because online interventions have relevance for people who have physical limitations or are not near facilities that could offer face-to-face interventions. Health behavior change interventions are relevant for cancer survivors, so continuing to test and refine interventions is imperative in the area of cancer survivorship."

21-ii) Discuss if there were elements in the RCT that would be different in a routine application setting

This was not discussed.

22) CONSORT: Interpretation consistent with results, balancing benefits and harms, and considering other relevant evidence

22-i) Restate study questions and summarize the answers suggested by the data, starting with primary outcomes and process outcomes (use)

"Participants in the treatment condition had significant reductions in insomnia and engaged in more strenuous and stretching exercises than those in the control condition. There is an established link between sleep disturbance and inflammation, which can be related to both cancer and depression [34], so impacting insomnia is a relevant finding. There is only one other known online exercise and diet intervention for adult cancer survivors [35]. Although outcomes of that study are not yet available, we have found the current system usable and the intervention feasible. In regards to face-to-face interventions to impact exercise for cancer survivors, these have been demonstrated to be effective [36-38], often times with larger effect sizes than were demonstrated in this trial. This is crucial because online interventions have relevance for people who have physical limitations or are not near facilities that could offer face-to-face interventions. Health behavior change interventions are relevant for cancer survivors, so continuing to test and refine interventions is imperative in the area of cancer survivorship."

22-ii) Highlight unanswered new questions, suggest future research

"Health behavior change interventions are relevant for cancer survivors, so continuing to test and refine interventions is imperative in the area of cancer survivorship."

"This leads to the question of whether we recruited survivors who were in need of support to improve multiple unhealthy habits, as well as whether this is one of the primary reasons that significant changes were not seen on many of the outcomes of interest (fatigue, depression, and increases in fruit and vegetable intake). The participants could also choose the behavior they wanted to change, regardless of their baseline level of that behavior or “need” to improve it. These factors could have contributed to the lack of significant change over the six-month period on some of the other outcome measures. In addition, when doing a population study where people enter with different concerns and a large range of scores on baseline measures, effect sizes can be muted. With a larger sample size, sensitivity analyses including only people who were not engaging in the health behaviors of interest at baseline could be explored. Future research could take into consideration these issues."

"Another potential limitation is in regards to the lack of participants with a range of cancer types. As has been the case in the past and was the case with our study, the sample included a large percentage of female breast cancer survivors (83% of the sample), suggesting that the sample was more homogenous and perhaps the findings are less generalizable to people with other types of cancer. Future efforts for this to be more balanced are important and will be made in upcoming work. Although efforts were made to recruit people who would be more representative of cancer survivors as a whole in regards to gender, ethnic, and cancer type, those efforts fell short in this study, and continued efforts will be made."

"Social networking features are often included in online interventions, such as the current intervention. Understanding more about things like who people interacted with, as well as the content of those interactions, provides a foundation to more fully understand the ways in which people connect and how those connections matter in these sorts of interventions. Continued inclusion of social networking/support in these types of interventions, as well as data collection on usage, is encouraged. Better understanding how the components included are used could also be a way to identify potent features of the intervention. It is important to note, though, that there could be synergistic effects that are tough to capture technically when isolating components of interest. In conclusion, the Thriving and Surviving with Cancer intervention has been proven a relative success, and additional efforts to understand what components are related to the most success could help further develop this, or any, online intervention program."

Other information

23) CONSORT: Registration number and name of trial registry

Clinical Trials.gov Identifier: NCT00962494

24) CONSORT: Where the full trial protocol can be accessed, if available

All IRB documentation is held with study personnel.

25) CONSORT: Sources of funding and other support (such as supply of drugs), role of funders

Department of Defense W81XWH-06-2-0042, Developmental Cancer Research Award from Stanford Cancer Center.

X26-i) Comment on ethics committee approval

"Participants were directed to the website and screened for eligibility and then completed an on-line consent approved by the University of Hawaii and Stanford IRBs. Participants from TAMC completed a mailed consent form that was approved by the military IRB."

x26-ii) Outline informed consent procedures

"Participants were directed to the website and screened for eligibility and then completed an on-line consent approved by the University of Hawaii and Stanford IRBs. Participants from TAMC completed a mailed consent form that was approved by the military IRB."

X26-iii) Safety and security procedures

Although this was discussed in the consenting process this was not discussed in the manuscript.

X27-i) State the relation of the study team towards the system being evaluated

Although this is not included in the manuscript, Stanford University does have a copyright on this program.
